# Virtual Screening of Cathelicidin-Derived Anticancer Peptides and Validation of Their Production in the Probiotic *Limosilactobacillus fermentum* KUB-D18 Using Genome-Scale Metabolic Modeling and Experimental Approaches

**DOI:** 10.3390/ijms262010077

**Published:** 2025-10-16

**Authors:** Vichugorn Wattayagorn, Taratorn Mansuwan, Krittapas Angkanawin, Chakkapan Sapkaew, Chomdao Sinthuvanich, Nisit Watthanasakphuban, Pramote Chumnanpuen

**Affiliations:** 1 Department of Biochemistry, Faculty of Science, Kasetsart University, Bangkok 10900, Thailand; vichugorn.wat@ku.th (V.W.); fscicds@ku.ac.th (C.S.); 2 Department of Zoology, Faculty of Science, Kasetsart University, Bangkok 10900, Thailand; taratorn.ma@ku.th (T.M.); krittapas.an@ku.th (K.A.); 3 Emarica Academy, Bangkok 10900, Thailand; chakkapan.s@ku.th; 4 Department of Biotechnology, Faculty of Agro-Industry, Kasetsart University, Bangkok 10900, Thailand; 5 Kasetsart University International College (KUIC), Kasetsart University, Bangkok 10900, Thailand

**Keywords:** genome-scale metabolic modeling, bioinformatics, virtual screening, anticancer peptides, colon cancer

## Abstract

The development of anticancer peptides (ACPs) has emerged as a promising strategy in targeted cancer therapy due to their high specificity and therapeutic potential. Cathelicidin-derived antimicrobial peptides represent a particularly attractive class of ACPs, yet systematic evaluation of their anticancer activity remains limited. In this study, we conducted virtual screening of eight cathelicidin-derived peptides (AL-38, LL-37, RK-31, KS-30, KR-20, FK-16, FK-13, and KR-12) to assess their potential against colon cancer. Among these, LL-37 and FK-16 were identified as the most promising candidates, with LL-37 exhibiting the strongest inhibitory effects on both non-metastatic (HT-29) and metastatic (SW-620) colon cancer cell lines in vitro. To overcome challenges associated with peptide stability and delivery, we employed the probiotic lactic acid bacterium *Limosilactobacillus fermentum* KUB-D18 as both a biosynthetic platform and delivery vehicle. A genome-scale metabolic model (GEM), *i*TM505, was reconstructed to predict the strain’s biosynthetic capacity for ACP production. Model simulations identified trehalose, sucrose, maltose, and cellobiose as optimal carbon sources supporting both high peptide yield and biomass accumulation, which was subsequently confirmed experimentally. Notably, *L. fermentum* expressing LL-37 achieved a growth rate of 2.16 gDW/L, closely matching the model prediction of 1.93 gDW/L (accuracy 89.69%), while the measured LL-37 concentration (26.96 ± 0.08 µM) aligned with predictions at 90.65% accuracy. The strong concordance between in silico predictions and experimental outcomes underscore the utility of GEM-guided metabolic engineering for optimizing peptide biosynthesis. This integrative approach—combining virtual screening, genome-scale modeling, and experimental validation—provides a robust framework for accelerating ACP discovery. Moreover, our findings highlight the potential of probiotic-based systems as effective delivery platforms for anticancer peptides, offering new avenues for the rational design and production of peptide therapeutics.

## 1. Introduction

Antimicrobial peptides (AMPs) such as AL-38, LL-37, RK-31, KS-30, KR-20, FK-16, FK-13, and KR-12 represent a unique class of bioactive molecules integral to the innate immune system. They exhibit potent antimicrobial activities chiefly by disrupting microbial membranes [1,2,3]. Among these, the Cathelicidin antimicrobial peptide (LL-37) and its derived fragments (KR-12, KR-20, FK-13, and FK-16) are particularly noteworthy due to their broad-spectrum efficacy against bacteria, fungi, and parasites, as well as their immunomodulatory functions [4,5]. Beyond their antimicrobial roles, these peptides have garnered significant attention for their emerging anticancer properties, placing them at the intersection of anti-infective and anticancer therapy. Anticancer peptides (ACPs) share structural similarities with AMPs—such as cationic charge and amphipathic α-helical or β-sheet conformations—that enable selective targeting of negatively charged cancer cell membranes, inducing apoptosis or necrosis while sparing normal cells [6,7]. LL-37 is an 18 kDa anti-microbial peptide in the Cathelicidin peptide group that is a human host defense molecule [8]. LL-37 interacts with the cell membrane, forming conducting channels that lead to the breakdown of the transmembrane potential [9]. LL-37 has the ability to disrupt the cancer cell membrane via toroidal pore models [10]. Previous studies have shown that LL-37 binds to the epidermal growth factor receptor (EGFR), thereby upregulating the tumor suppressor gene p53, which regulates cell division. LL-37 inhibits the growth of HT-29 and HCT116 colon cancer cell lines and induces apoptosis [11]. Peptides like LL-37 and its derivatives exhibit a capacity to inhibit tumor growth, induce cell death mechanisms, and modulate immune responses, making them promising candidates for novel anticancer therapeutics [2,3,5]. Despite challenges in stability and delivery, ongoing research aims to optimize these dual-function peptides for clinical applications, highlighting their potential as versatile agents against both infectious diseases and cancer. This overview underscores the therapeutic promise of these peptides as antimicrobial and anticancer agents, reflecting their multifunctional bioactivity and significant biomedical relevance [5,8].

Metabolic engineering is a discipline that strategically modifies cellular metabolic pathways to enhance the production of target metabolites. This strategy has been widely applied to increase product yields and reduce the costs associated with the biosynthesis of biological compounds, including peptides. Cellular systems are highly dynamic, responding to diverse stimuli and orchestrating complex biochemical processes. Consequently, mathematical modeling plays a pivotal role in advancing our understanding of cellular metabolism [12]. Genome-scale metabolic models (GEMs) are predictive tools of high accuracy, designed under well-defined conditions and tailored to specific applications [13].

Currently, protein expression systems in probiotics have been extensively developed. Probiotics play an important role in food applications and have been engineered as cell factories for inducible protein expression, as well as serving as live therapeutic protein-delivery vehicles. Recent studies have focused on the application of the probiotic *Limosilactobacillus fermentum* KUB-D18. This strain exhibits multiple beneficial properties, including inhibition of intestinal pathogens, enhancement of immunity, promotion of digestion, and anti-inflammatory effects [14]. The incidence of colorectal cancer has been rising rapidly, currently ranking as the third most common cancer worldwide [15,16]. Standard treatment options include chemotherapy, surgery, and radiotherapy; however, the efficacy of radiotherapy is limited by factors such as disease stage, tumor size, and the severity of treatment-related side effects. Consequently, the discovery and development of alternative therapeutic strategies remain a major challenge in modern medicine.

This study aims to integrate virtual screening with experimental validation to evaluate the anticancer potential of cathelicidin-derived peptides. Moreover, this study illustrates the potential application of LL-37 and its derived fragments as targeted inhibitors of colorectal cancer, offering high specificity with minimal side effects. Peptide delivery is achieved through genetically engineered probiotics, with genome-scale metabolic modeling (GEM) employed to predict optimal culture conditions for LL-37 production. Furthermore, insights obtained from the GEM, combined with the development of LL-37 delivery systems using *Limosilactobacillus fermentum* KUB-D18, are expected to advance medical technology and support the development of alternative therapeutic strategies, thereby contributing to progress in medical and pharmaceutical sciences.

## 2. Results and Discussion

### 2.1. Comparative Functional Analysis of Eight Cathelicidin-Derived Anticancer Peptides

**Figure 1:**
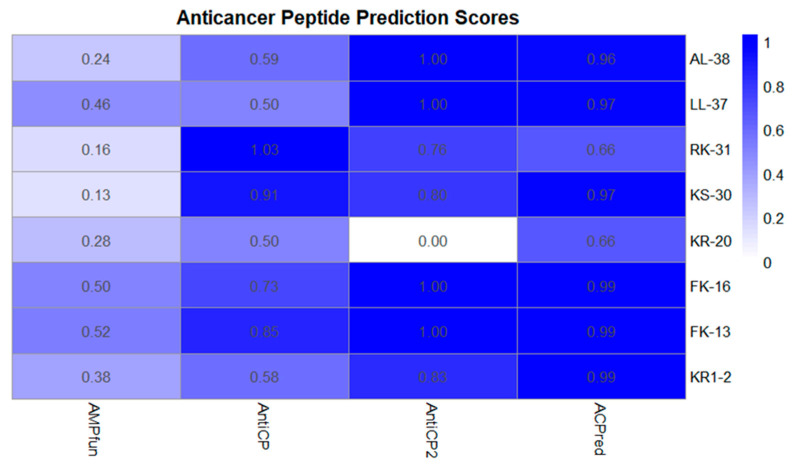
Prediction analysis of anticancer peptides using AMPfun, AntiCP, AntiCP2.0, and ACPred, classified according to probabilistic scores and predicted functions.

**Figure 2:**
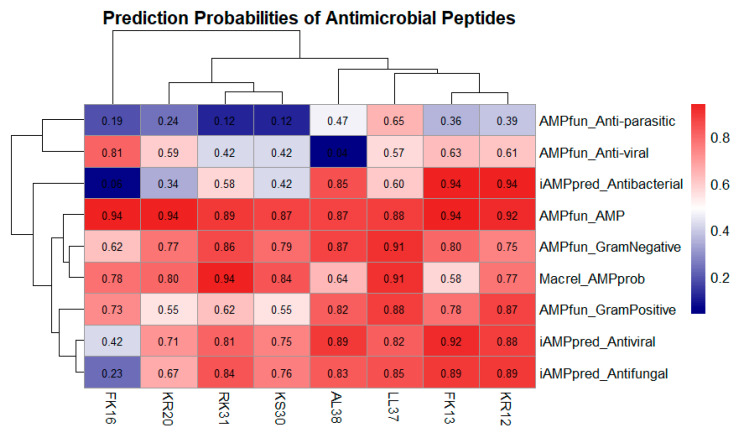
Prediction analysis of antibacterial and antimicrobial activities using AMPfun, iAMPpred, and Macrel, classified according to probabilistic scores and predicted functions.

Eight peptides, including AL-38, LL-37, RK-31, KS-30, KR-20, FK-16, FK-13, and KR-12 (Table 1), were computationally evaluated for their anticancer potential using multiple online databases. The scoring system employed a normalized sigmoid scale ranging from 0 (low probability of biological activity) to 1 (high probability of biological activity). Anticancer properties were predicted using AMPfun, AntiCP, AntiCP2.0, and ACPred (Figure 1). Potential side effects were systematically evaluated, as the proposed delivery system relies on a probiotic bacterial cell factory. In addition to anticancer potential, antimicrobial and antibacterial activities were assessed to ensure that high peptide expression would not compromise the growth or stability of the host probiotic. Antibacterial activity was analyzed using AMPfun, iAMPpred, and Macrel (Figure 2).

All eight peptides exhibited PROB scores below 0.5 in HAPPENN, indicating minimal hemolytic potential. Allergenicity predictions using AllerTop, AllergenFP, and AllerCatPro suggested that LL-37, KR-20, FK-16, FK-13, and KR-12 are non-allergenic (Table 2). Cytotoxicity was assessed using ToxinPred and ToxIBTL (Table 3), with the support vector machine (SVM) output in ToxinPred showing negative values for all peptides, indicating a low likelihood of toxicity to mammalian cells. Hemolytic activity was further evaluated using HAPPENN, HemoPred, and Macrel (Table 4).

Collectively, these computational predictions supported the selection of LL-37 for further development. Its dual profile of strong anticancer potential combined with minimal predicted cytotoxicity, hemolysis, and allergenicity renders it a suitable candidate for delivery via genetically engineered probiotics. Furthermore, genome-scale metabolic modeling of *Limosilactobacillus fermentum* KUB-D18 provides a robust framework to optimize peptide production and delivery strategies.

Computational prediction of anticancer activity was conducted using four independent web servers (AMPfun, AntiCP, AntiCP 2.0, and ACPred), providing complementary yet distinct insights into the potential of the evaluated peptides. AMPfun indicated that only FK-16 (0.50) and FK-13 (0.52) achieved moderate scores, whereas most peptides exhibited relatively low probabilities (<0.40), suggesting a stricter threshold for anticancer classification. In contrast, the SVM-based AntiCP classified all peptides as anticancer, with RK-31 and KS-30 attaining the highest scores (1.03 and 0.91, respectively). AntiCP 2.0 further confirmed high anticancer probabilities for most peptides, including AL-38, LL-37, FK-16, FK-13, and KR1-2 (scores ≥ 0.80). Notably, KR-20 was predicted as non-anticancer by AntiCP 2.0, despite showing borderline probabilities in other tools. ACPred consistently classified all peptides as anticancer with high confidence (≥0.96 for most, except RK-31 and KR-20, both 0.66), while assigning extremely low scores for the non-anticancer category (<0.05 for most peptides).

Collectively, these results indicate that FK-16, FK-13, and LL-37 are among the most promising candidates for anticancer activity, as they consistently achieved high scores across all prediction platforms. These findings are in agreement with previous studies reporting cytotoxicity of FK-16 and LL-37 fragments against colon and gastric cancer cell lines through mechanisms involving membrane disruption, apoptosis induction, and immune modulation. RK-31 and KS-30 also demonstrated favorable predictions in AntiCP-based models, suggesting that structural features such as cationicity and amphipathicity may underlie their anticancer potential. The discrepancy observed for KR-20—classified as non-anticancer by AntiCP 2.0 but predicted as anticancer by other platforms—highlights the necessity of experimental validation, as in silico predictions are inherently model-dependent and sensitive to training datasets.

Overall, integration of results from multiple prediction servers suggests that several peptides, particularly FK-16, FK-13, and LL-37, hold significant promise as anticancer peptides (ACPs). Their consistent cross-platform predictions, supported by literature evidence, identify them as strong candidates for further experimental validation in cancer cell line assays to confirm their therapeutic potential.

Accumulated evidence strongly supports the dual functionality of several antimicrobial peptides (AMPs), including AL-38, LL-37, RK-31, KS-30, KR-20, FK-16, FK-13, and KR-12, which exhibit both antimicrobial and anticancer activities. Felício et al. (2017) demonstrated that these peptides are active against a wide spectrum of cancer cell lines, including drug-resistant breast cancer cells, while retaining antimicrobial effects [17]. Similarly, Gaspar et al. (2013) reviewed the broad-spectrum anticancer potential of these peptides across multiple carcinoma types—including breast, prostate, melanoma, colon, lung, and leukemia—reflecting a conserved functional motif that confers selective cytotoxicity toward malignant cells [6].

### 2.2. Validation of LL-37 and FK-16 on Colon Cancer Cell Lines

**Figure 3:**
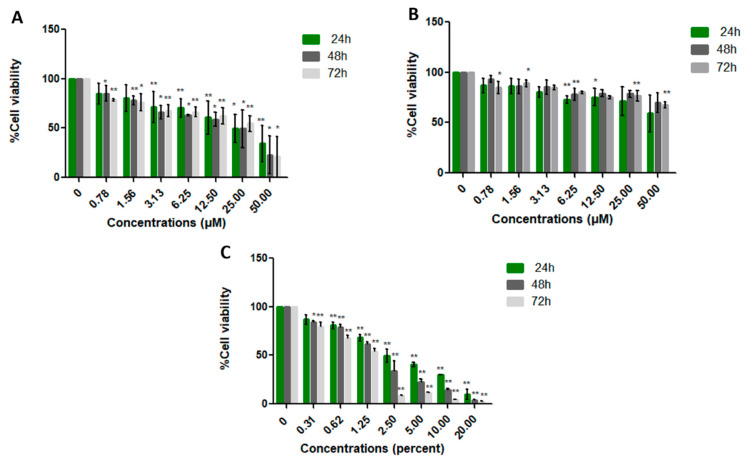
Cytotoxicity of LL-37 (**A**), FK-16 (**B**), and DMSO (**C**) on the metastatic colon cancer cell line SW-620, assessed by MTT assay at 24, 48, and 72 h. Cell viability was expressed relative to untreated controls (0 µM) and presented as mean ± SD. Statistical analysis was performed using Student’s *t*-test (*, *p* ≤ 0.05; **, *p* ≤ 0.01).

**Figure 4:**
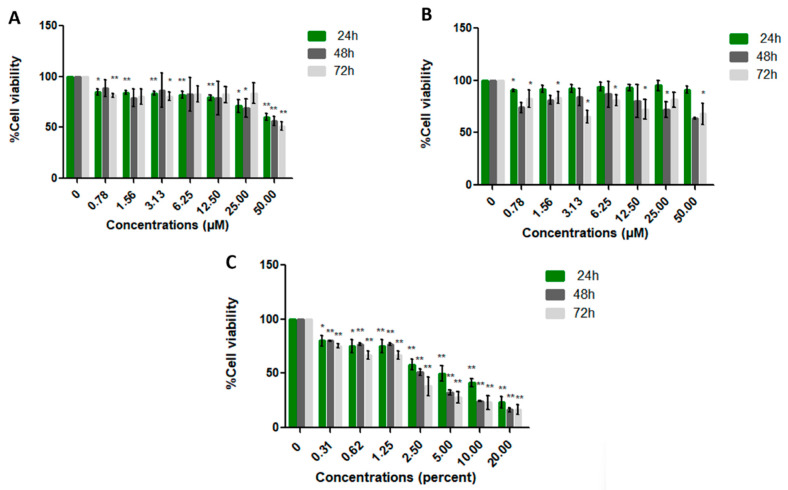
Cytotoxicity of LL-37 (**A**), FK-16 (**B**), and DMSO (**C**) on the non-metastatic colon cancer cell line HT-29, assessed by MTT assay at 24, 48, and 72 h. Cell viability was expressed relative to untreated controls (0 µM) and presented as mean ± SD. Statistical analysis was performed using Student’s *t*-test (*, *p* ≤ 0.05; **, *p* ≤ 0.01).

**Figure 5:**
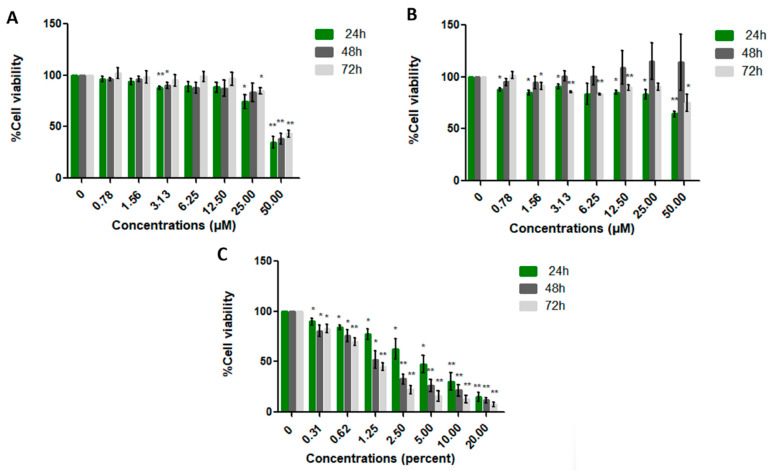
Cytotoxicity of LL-37 (**A**), FK-16 (**B**), and DMSO (**C**) on the normal human fibroblast cell line PCS-201-010, assessed by MTT assay at 24, 48, and 72 h. Cell viability was expressed relative to untreated controls (0 µM) and presented as mean ± SD. Statistical analysis was performed using Student’s *t*-test (*, *p* ≤ 0.05; **, *p* ≤ 0.01).

Cytotoxicity of cancer and normal cell lines was evaluated using the MTT assay. Cells were treated with varying concentrations of LL-37 and FK-16 (0–50 µM), as well as the positive control (DMSO, 0–20%), for 24, 48, and 72 h. In this study, we employed a human normal fibroblast cell line (PCS-201-010) together with two human colon cancer cell lines: HT-29 (non-metastatic) and SW-620 (metastatic). These cancer cell lines were selected because they differ in both intracellular mechanisms and morphological characteristics. By comparing their responses, we aimed to determine whether the peptides exert stronger inhibitory effects on non-metastatic or metastatic cells, thereby providing valuable insights for the development of more targeted therapeutic strategies. The MTT assay evaluates the activity of mitochondrial enzymes in live cells following treatment. The half-maximal inhibitory concentration (IC_50_) values of LL-37 for SW-620 cells were 21.42 ± 11.44, 15.28 ± 6.67, and 17.33 ± 8.20 µM at 24, 48, and 72 h, respectively. LL-37 significantly inhibited SW-620 cell proliferation in a dose-dependent manner relative to the control and other cell lines (Figure 3, Figure 4 and Figure 5). Comparative analysis of IC_50_ values indicated that LL-37 exhibited greater anticancer activity than FK-16 (Table 5). FK-16 at 50 µM did not achieve sufficient inhibition to determine an IC_50_ value. Consequently, LL-37 was selected for recombinant plasmid construction to further evaluate its production potential in probiotic hosts.

Mechanistically, antimicrobial peptides (AMPs) such as LL-37 and its derivatives have been demonstrated to exert anticancer effects primarily through membrane disruption, leading to tumor cell lysis, and by promoting the release of tumor antigens, which may enhance immune recognition [18,19,20]. Notably, studies on LL-37 fragments have also revealed antibacterial and antibiofilm activities against multidrug-resistant pathogens, including *Acinetobacter baumannii*, underscoring their potential for dual applications in oncology and infectious disease control [21]. The use of peptide-linked delivery systems, such as PLGA-conjugate micelles, further enhances the therapeutic index of LL-37 analogs by improving stability and cellular uptake in tumor cells, as demonstrated by Mori et al. (2021) [22]. Prior studies have evaluated these peptides across an extensive panel of cancer cell lines—including colon carcinoma (HCT-116, HT-29), melanoma (B16/BL6), leukemia (Jurkat, U937), hepatocellular carcinoma (HepG2, Huh-7, SMMC-7721), breast carcinoma (MCF-7, MDA-MB-435S), and lung carcinoma (A549, H460)—highlighting their versatility in targeting diverse tumor types. This broad-spectrum activity suggests that conserved physicochemical features, such as cationicity, amphipathicity, and the ability to interact with negatively charged membranes, play a critical role in their anticancer mechanisms.

Collectively, the literature reinforces our computational predictions for LL-37 and its derivatives, particularly FK-16, supporting their selection for further development. Their dual antimicrobial–anticancer profile, combined with minimal predicted cytotoxicity toward normal cells, low hemolytic potential, and absence of allergenicity, positions them as promising candidates for delivery via genetically engineered probiotic systems. Moreover, integration of genome-scale metabolic modeling of *Limosilactobacillus fermentum* KUB-D18 provides a rational framework for optimizing peptide production while mitigating potential inhibitory effects on the probiotic host.

### 2.3. Characteristics of the Genome-Scale Metabolic Model of L. fermentum KUB-D18 (iTM505)

To investigate the optimal conditions for growth and LL-37 peptide production in *L. fermentum* KUB-D18, a genome-scale metabolic model (GEM) of the bacterium was constructed and analyzed. The model predicts relevant metabolic pathways and evaluates cellular performance under diverse environmental conditions. Genomic sequence data were retrieved from the National Center for Biotechnology Information (NCBI) database and used to identify enzyme–gene–metabolite associations via the ModelSEED platform, which facilitated reconstruction of the bacterial metabolic network. The resulting GEM, designated *i*TM505, comprises 505 genes, 1191 metabolites, and 1095 reactions, distributed across two cellular compartments: the cytoplasm and the extracellular space (Table 6).

**Figure 6:**
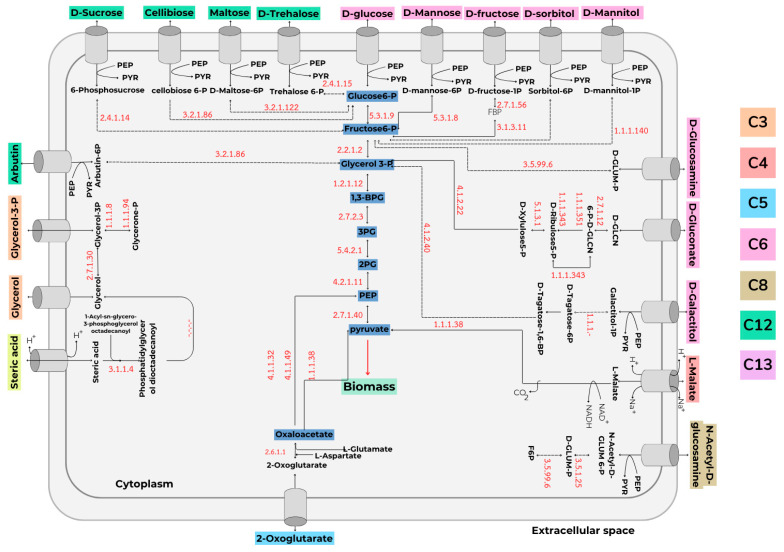
Schematic representation of carbon source transport pathways in *L. fermentum* KUB-D18 (*i*TM505). Dashed lines indicate reactions supplemented based on ModelSEED data.

In addition, the *i*TM505 metabolic model identified a total of 19 carbon sources that *L. fermentum* KUB-D18 can utilize for growth and the production of metabolites. These carbon sources range from compounds containing 3, 4, 5, 6, 8, 12, and 13 carbon atoms. All of seven carbon sources can enter the cell through the glycolysis metabolic pathway. Monosaccharide sugars can enter the glycolysis pathway through fructose-6-phosphate. Disaccharide sugars enter the cell that catalase by enzymes into monosaccharides. Additionally, five-carbon sugars enter the cell and are converted into α-ketoglutarate, subsequently entering the metabolic glycolysis pathway via phosphoenolpyruvate. Other carbon sources can also enter the metabolic glycolysis pathway similarly to contribute to biomass formation (Figure 6).

### 2.4. Identification of Nutrient Sources Essential for the Growth of L. fermentum KUB-D18 and Enhancement of Growth Performance Under Simulation Using the iTM505 Model in Various Limited Carbon Source Conditions

**Figure 7:**
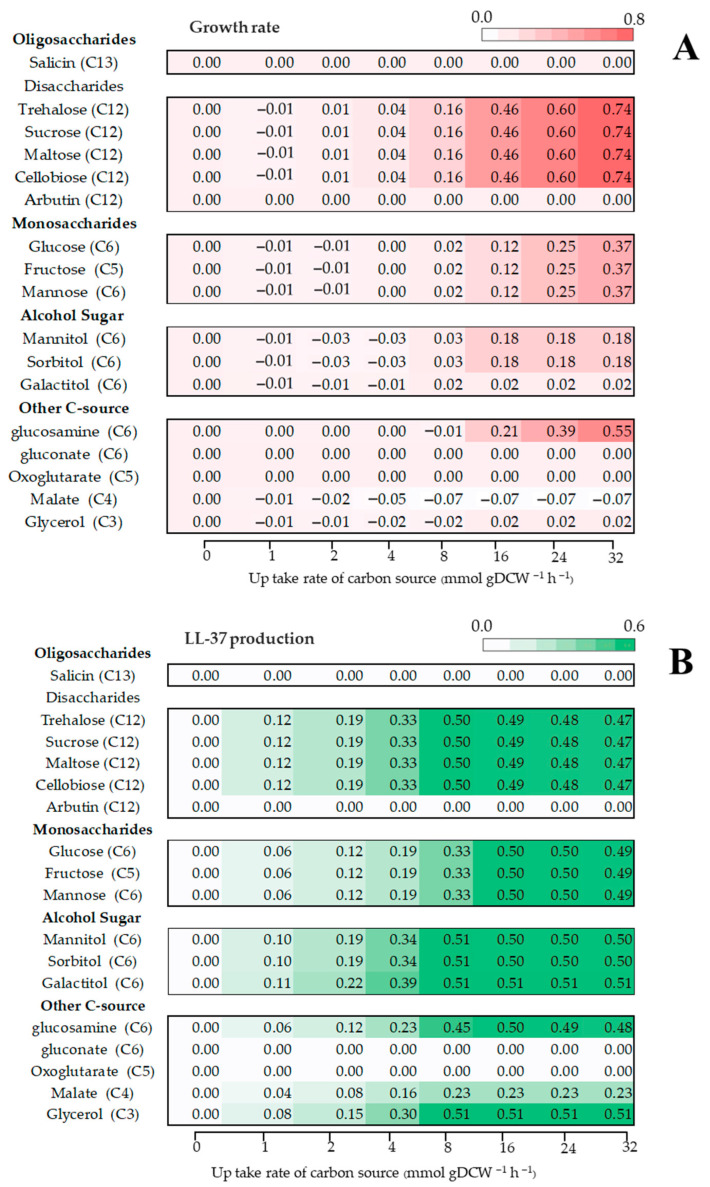
Predicted growth rates (**A**) and LL-37 production (**B**) of *L. fermentum* KUB-D18 under various carbon sources. Individual values within the matrices indicate predicted growth rates corresponding to different carbon uptake rates. The red color scale denotes biomass prediction (**A**), while the green color scale represents LL-37 peptide production (**B**).

The *i*TM505 genome-scale metabolic model was analyzed under various carbon-limited conditions, including oligosaccharides (salicin), monosaccharides (glucose, fructose, and mannose), disaccharides (trehalose, sucrose, maltose, cellobiose, and arbutin), alcohol sugars (mannitol, sorbitol, and galactitol), and other carbon sources (glucosamine, gluconate, oxoglutarate, malate, and glycerol). These carbon sources were selected based on the presence of corresponding transporter genes identified in the annotated genome of *L. fermentum* KUB-D18. Nevertheless, model simulations indicated that some carbon sources were not efficiently utilized by the cells or did not significantly contribute to biomass formation or LL-37 production (Figure 7).

**Figure 8:**
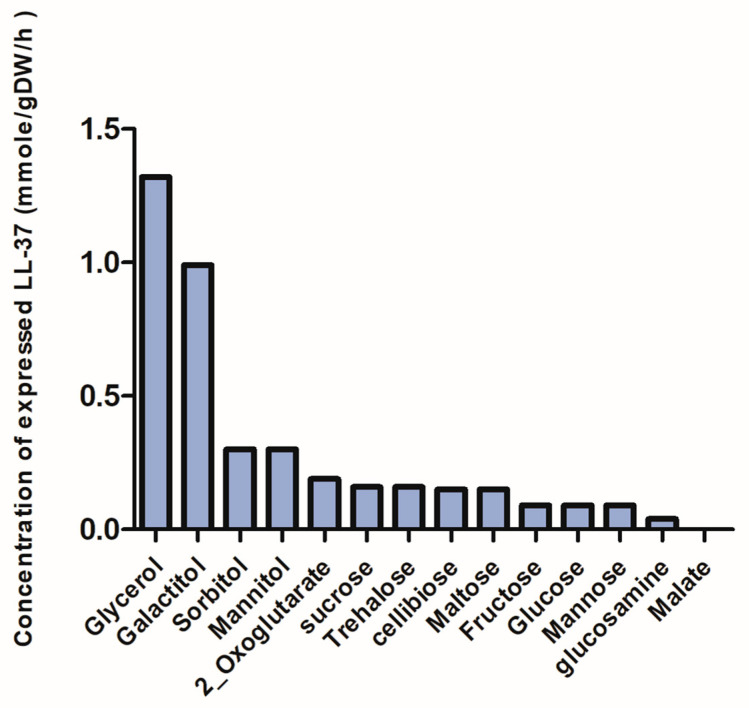
LL-37 peptide production for various carbon sources based on the *i*TM505 model simulation.

The results demonstrated that disaccharides supported higher predicted growth rates of *L. fermentum* KUB-D18 compared with other sugars when evaluated at equivalent carbon uptake rates (mmol gDCW^−1^ h^−1^). The maximum predicted growth rate was 0.74 h^−1^ at 32 h. Regarding LL-37 peptide biosynthesis under various carbon-limited conditions, alcohol sugars (glycerol and galactitol) supported the highest predicted LL-37 production (Figure 8). Nevertheless, the predicted yields for disaccharides and monosaccharides were comparable (Figure 7 and Figure 8).

This study demonstrates the development of probiotics as a cell factory to produce anticancer peptides. LL-37 was selected for recombinant plasmid construction based on in silico prediction results. Previous studies have indicated that LL-37 possesses a positive charge and hydrophobicity, enabling interaction with negatively charged cancer cell membranes. Accordingly, LL-37 can disrupt cancer cell membranes via toroidal pore formation, one of the established mechanisms of cationic antimicrobial peptides (AMPs) [10]. *Limosilactobacillus fermentum* KUB-D18, a probiotic isolated from chicken intestines, was chosen as the expression host and delivery vehicle for LL-37. This strain grows optimally in de Man, Rogosa, and Sharpe (MRS) medium at 30–40 °C [23] and exhibits rapid and robust growth. The efficiency of peptide expression is strongly influenced by the culture medium. MRS medium is specifically formulated to support Lactobacillus growth and comprises: (1) proteose peptone as a source of nitrogen-containing compounds; (2) beef or meat extract as additional carbon and nitrogen sources; (3) yeast extract providing essential vitamins (B-complex) and amino acids; (4) dextrose (glucose) as the primary carbohydrate source; (5) polysorbate 80 (Tween 80) to facilitate nutrient uptake; (6) sodium acetate to inhibit Streptococci and molds while promoting Lactobacillus growth; (7) ammonium citrate to suppress unwanted microorganisms and supply nutrients; (8) magnesium sulfate and manganese sulfate as essential ions for metabolic processes; and (9) dipotassium hydrogen phosphate or other phosphates to maintain pH stability [24].

In this study, *L. fermentum* KUB-D18 was intended to produce the LL-37 peptide in a medium compatible with colon cancer cell culture, such as RPMI 1640 (Roswell Park Memorial Institute Medium). RPMI 1640 is primarily formulated for mammalian cells, including human leukemic cells, lymphocytes, and various adherent and suspension cell types. The medium contains amino acids (e.g., L-glutamine, L-arginine) essential for cellular growth, B-complex vitamins (B12, B6, folic acid) to support metabolic processes, glucose as the primary energy source, and minerals (NaCl, KCl, MgSO_4_, CaCl_2_) to maintain electrolyte balance. Sodium bicarbonate (NaHCO_3_) is included to regulate pH, and additional supplements such as HEPES or sodium pyruvate may be added to optimize the culture environment [25]. However, RPMI 1640 is not ideally suited for supporting peptide expression by *L. fermentum* KUB-D18. Although fetal bovine serum (FBS) contains amino acids that facilitate protein synthesis and has been developed as a supplement for eukaryotic cell culture, it can also promote bacterial growth under certain conditions. For instance, the addition of 10% FBS to Brucella broth has been reported to enhance biofilm formation by *Helicobacter pylori* [26]. Nevertheless, the presence of FBS in combination with LL-37 treatment influenced colon cancer cell viability, resulting in inconsistent and potentially inaccurate IC_50_ values obtained from the MTT assay.

### 2.5. Expression of LL-37 from L. fermentum KUB-D18

**Figure 9:**
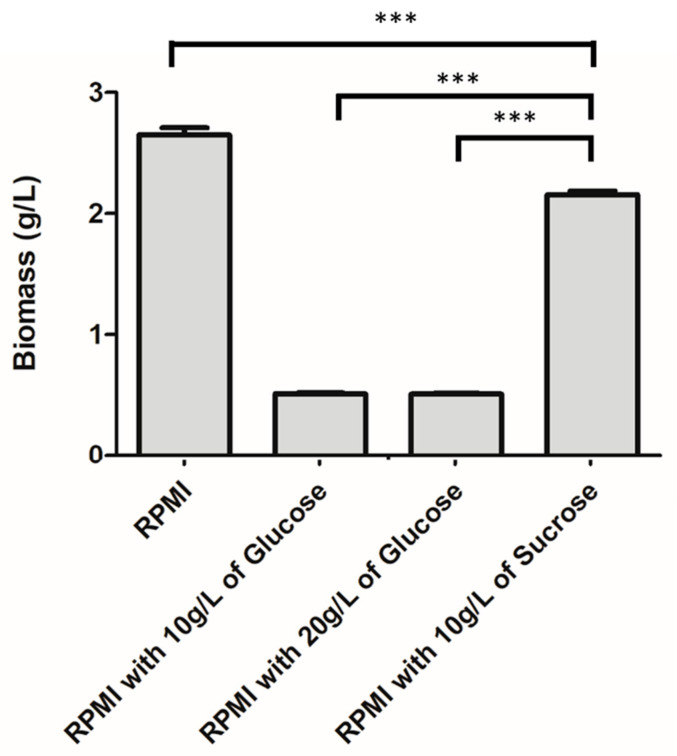
Biomass of *L. fermentum* KUB-D18 harboring pSIP411 + LL-37 cultured under different media conditions at 30 °C for 24 h. Data are presented as mean ± SD. Statistical significance was assessed using Tukey’s multiple comparison test (***, *p* ≤ 0.001).

The recombinant plasmids, pSIP411 + LL-37, were transformed into *L. fermentum* KUB-D18. This study aimed to determine optimal conditions for LL-37 peptide production suitable for potential applications in colon cancer cell treatment and to identify conditions compatible with animal cell culture media. Biomass measurements revealed that *L. fermentum* KUB-D18 achieved 2.166 g/L in RPMI medium supplemented with 10 g/L sucrose, which differed significantly from other conditions: RPMI medium alone (2 g/L glucose) at 2.730 g/L, RPMI with 10 g/L glucose (total 12 g/L) at 0.503 g/L, and RPMI with 20 g/L glucose (total 22 g/L) at 0.510 g/L (Figure 9). In comparison, the *i*TM505 genome-scale metabolic model predicted a biomass of 1.933 g/L under RPMI medium with 10 g/L sucrose, corresponding to 89.69% accuracy relative to experimental data.

**Figure 10:**
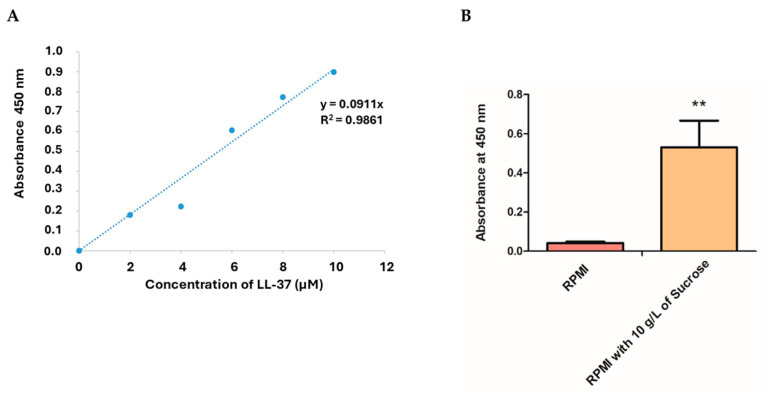
ELISA quantification of LL-37 peptide expression by *L. fermentum* KUB-D18 harboring pSIP411 + LL-37. (**A**) Standard curve generated using synthetic LL-37 peptide for quantification. (**B**) Comparison of LL-37 expression levels in RPMI medium alone and RPMI supplemented with 10 g/L sucrose. Data are presented as mean ± SD. Statistical significance was assessed using one-way ANOVA (**, *p* ≤ 0.01).

**Figure 11:**
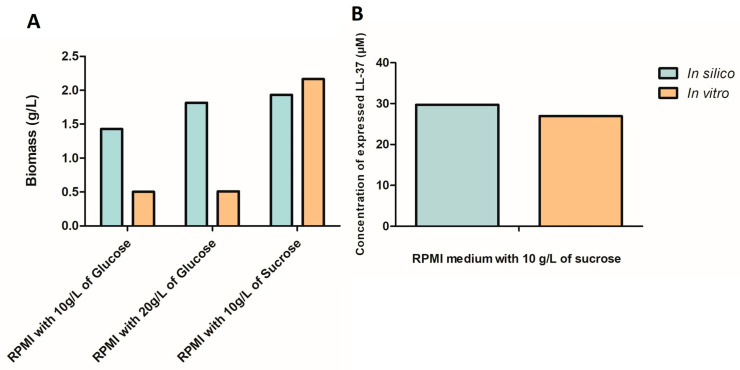
Validation of the *i*TM505 genome-scale metabolic model by comparison of (**A**) biomass and (**B**) LL-37 peptide expression between in silico predictions and in vitro measurements across different carbon sources.

Indirect competitive ELISA indicated that *L. fermentum* KUB-D18 harboring pSIP411+ LL-37 expressed higher LL-37 levels in RPMI medium supplemented with 10 g/L sucrose than in RPMI medium alone. The *i*TM505 model predicted LL-37 production at 0.16 mmole/gDW/h under this condition, corresponding to approximately 29.73 µM based on a molecular weight of 5.38 kDa. This prediction closely matched the experimental ELISA quantification of 26.96 ± 0.08 µM, yielding an accuracy of 90.65% (Figure 10 and Figure 11).

The application of genome-scale metabolic modeling (GEM) is highly valuable for optimizing experimental conditions in this study. Using the *i*TM505 model of *L. fermentum* KUB-D18, a total of 19 carbon source transport systems were identified, enabling the import and export of various carbon substrates. These results are consistent with findings reported by Yuke He (2025) [27]. The carbohydrate transport system diagram revealed three additional transport systems for raffinose, galactose, and lactose. However, the *i*TM505 model did not identify the genes responsible for transporting these sugars into the cell, which differs from the observations reported by Yuke He (2025) [27]. The *i*TM505 model was further analyzed under carbon-limited conditions, both with and without the LL-37 peptide biosynthetic reaction. In both scenarios, the highest predicted biomass production was achieved with disaccharides, including trehalose, sucrose, maltose, and cellobiose. During glycolysis, these disaccharides are hydrolyzed into monosaccharides and subsequently converted into pyruvate. The model predictions indicate that these carbon substrates are primarily metabolized via the phosphotransferase system (PTS), the main sugar transport mechanism in many Gram-positive and Gram-negative bacteria [28]. The genome of *L. fermentum* KUB-D18 encodes 11 PTS transporter genes, including those responsible for sucrose uptake and phosphorylation [27]. Sucrose is phosphorylated to sucrose-6-phosphate by PTS transporters and then hydrolyzed into glucose-6-phosphate and fructose, which enter glycolysis or heterofermentative pathways.

Analogously, sucrose uptake in *Streptococcus thermophilus* ASCC 1275 has been shown to enhance exopolysaccharide (EPS) production compared to glucose or lactose. In the food industry, EPS from lactic acid bacteria (LAB) starter cultures is used as a moisture-retention agent in cheese, improving texture and functionality [29].

Experimental validation of LL-37 expression in *L. fermentum* KUB-D18 corroborated the model predictions, showing elevated peptide levels in RPMI medium supplemented with sucrose compared to other media. The *i*TM505 model predicted LL-37 production at 0.16 mmol/gDW/h, corresponding to approximately 29.73 µM based on a molecular weight of 5.38 kDa, closely matching the ELISA-quantified concentration of 26.96 ± 0.08 µM, achieving 90.65% accuracy. This strong concordance demonstrates the robustness of the model in guiding experimental design.

Overall, the *i*TM505 model provides a predictive framework for selecting suitable carbohydrate sources to support the growth and productivity of *L. fermentum* KUB-D18. It enables the rational optimization of cultivation strategies to maximize both biomass yield and target metabolite production and may be further applied to predict how dietary components influence gut microbiota and immune responses.

### 2.6. Significance of the First GEM for L. fermentum KUB-D18 and Its Experimental Validation

In the context of the genus *Limosilactobacillus*, the majority of previous genome-scale metabolic modeling (GEM) efforts have been concentrated on *L. reuteri*. A notable example is the *i*TN656 model of *L. reuteri* KUB-AC5 [30], which demonstrated that disaccharides, particularly sucrose, provided superior support for bacterial growth compared to monosaccharides. The model further indicated that sucrose utilization in *L. reuteri* is facilitated through starch and sucrose metabolism, specifically involving sucrose phosphorylase (EC: 2.4.1.7), which catalyzes the phosphorolysis of sucrose to generate glucose 1-phosphate and fructose [31].

Our *i*TM505 model of *L. fermentum* KUB-D18 is consistent with these findings, as disaccharides—especially sucrose—were predicted and experimentally validated to be the most effective carbon sources for both biomass formation and LL-37 production. Importantly, the predictive accuracy of *iTM505* (90.65%) was higher than that reported for *iTN656*, highlighting the robustness and reliability of our model construction.

Beyond modeling, *L. fermentum* may also hold advantages over *L. reuteri* as a probiotic host. Several studies have highlighted its strain-specific benefits, including stronger anti-inflammatory effects in animal models of colitis (e.g., reduced COX-2 expression and prevention of glutathione depletion) [32], enhanced stimulation of short-chain fatty acid (SCFA) production such as butyrate [32,33], and potential contributions to managing metabolic disorders including diabetes and hypercholesterolemia (e.g., *L. fermentum* ME-3) [34,35]. Furthermore, certain strains of *L. fermentum* have been reported to support respiratory and immune health [36].

The present study represents the first attempt to construct and optimize a genome-scale metabolic model (GEM) of *L. fermentum* KUB-D18, a probiotic bacterium with the capacity for engineered peptide production. Developing such a GEM is inherently complex, requiring iterative steps of model curation, gap filling, parameter adjustment, and precise definition of the objective function. Despite the challenges, the model proved to be highly reliable in simulating bacterial growth under different carbon sources and in predicting the production potential of the LL-37 peptide.

The experimental validation through plasmid transformation and peptide secretion assays further strengthened the utility of the GEM. Achieving secretion of LL-37 into the culture medium is technically challenging, yet the experimental results correlated well with the in silico predictions, underscoring the accuracy and robustness of the model. This emphasizes the essential role of GEMs not merely as theoretical frameworks but as predictive tools that can guide experimental design with high confidence.

Moreover, the integration of GEMs into probiotic engineering demonstrates their broader applicability in synthetic biology. By predicting feasible and optimal conditions prior to experimentation, GEMs can minimize laborious trial-and-error processes, shorten experimental timelines, reduce overall costs, and eliminate impractical approaches. Thus, the combination of computational modeling with experimental validation creates a rational workflow that enhances both efficiency and innovation in research.

While this study provides the first version of a GEM for *L. fermentum* KUB-D18, it already functions effectively as a platform for further exploration. Researchers working in probiotic biotechnology and peptide delivery can build upon this foundation, adapting and refining the model for diverse applications. Rather than being regarded as “basic,” this initial GEM version is better viewed as a critical stepping stone—one that contributes substantially to the rapidly expanding field of synthetic biology and paves the way for advanced probiotic-based therapeutic strategies.

## 3. Materials and Methods

### 3.1. Bioinformatic Functional and Analysis

Eight anticancer peptide sequences were selected for analysis, including AL-38, LL-37, RK-31, KS-30, KR-20, FK-16, FK-13, and KR-12 (Table 1). The sequences were arranged in FASTA format and used as input for property prediction.

Anticancer activity was predicted using tools such as AMPfun, (http://crdd.osdd.net/raghava/anticp/, accessed on 9 November 2022), antiCP 2.0 (https://webs.iiitd.edu.in/raghava/anticp2/, accessed on 9 November 2022), and ACPred (http://codes.bio/acpred/, accessed on 9 November 2022). Antibacterial potential was assessed with AMPfun (http://fdblab.csie.ncu.edu.tw/AMPfun/, accessed on 9 November 2022), iAMPpred (http://cabgrid.res.in:8080/amppred/index/, accessed on 9 November 2022) and Macrel (https://www.big-data-biology.org/software/macrel/, accessed on 9 November 2022). ToxinPred (http://crdd.osdd.net/raghava/toxinpred/, accessed on 9 November 2022) and ToxIBTL (https://server.wei-group.net/ToxIBTL/, accessed on 9 November 2022) were employed to predict and design toxic or non-toxic peptides. Hemolytic activity was evaluated using HAPPENN (https://research.timmons.eu/happenn/, accessed on 9 November 2022), HemoPred (http://codes.bio/hemopred/, accessed on 9 November 2022) and Macrel (https://www.big-data-biology.org/software/macrel/, accessed on 9 November 2022). Allergenicity was predicted with AllerTop (https://www.ddg-pharmfac.net/AllerTOP/, accessed on 9 November 2022), AllergenFP (https://ddg-pharmfac.net/AllergenFP/, accessed on 9 November 2022) and AllerCatPro (https://allercatpro.bii.a-star.edu.sg/, accessed on 9 November 2022) based on key physicochemical properties of proteins.

### 3.2. Cell Culture

The normal human fibroblast cell line (PCS-201-010), the non-metastatic human colon cancer cell line (HT-29), and the metastatic human colon cancer cell line (SW-620) were kindly provided by Dr. Mattaka Khongkaw, Senior Researcher at the National Nanotechnology Center (NANOTEC), Thailand. PCS-201-010 and HT-29 cells were cultured in Dulbecco’s Modified Eagle’s Medium (DMEM; Gibco, Thermo Fisher, Waltham, MA, USA), whereas SW-620 cells were maintained in Roswell Park Memorial Institute Medium 1640 (RPMI 1640; Gibco, Thermo Fisher, USA). All media were supplemented with 10% fetal bovine serum (FBS) and 1% penicillin–streptomycin (Gibco, Thermo Fisher, USA). Cells were incubated at 37 °C in a humidified atmosphere containing 5% CO_2_ and 95% air. Trypsin–EDTA was used for cell detachment when cultures reached approximately 70% confluency.

*L. fermentum* KUB-D18 was kindly provided by Associate Professor Massalin Nakphaichit, Department of Biotechnology, Faculty of Agro-Industry, Kasetsart University, Thailand, and was cultivated in Man–Rogosa–Sharpe (MRS; Difco, Tucker, GA, USA) medium at 30 °C under static conditions.

### 3.3. Determination of Cell Viability by MTT Assay

The cytotoxicity of LL-37 and FK-16 was evaluated using the MTT assay. HT-29, SW-620, and PCS-201-010 cells were seeded at a density of 5 × 10^4^ cells/mL in 100 µL per well in 96-well plates and allowed to adhere for 24 h. On the following day, the cells were treated with varying concentrations of LL-37 (0–50 µM), FK-16 (0–50 µM), or the positive control DMSO (0–20%) for 24, 48, and 72 h. The peptides LL-37 and FK-16 used for validation on cell lines were obtained from ChinaPeptides (Hangzhou, China) with a reported purity of 95%. To evaluate peptide efficacy, 10 µL of 5 mg/mL MTT solution was added and incubated for 4 h at 37 °C to form formazan crystal. Next, the excess MTT solution was carefully removed and replaced with 100 µL of DMSO to dissolve the formazan crystals. Absorbance was measured at 570 nm using a microplate reader (BioTek, Winooski, VT, USA). The percentage of cell viability was quantified using the following formula, and the half-maximal inhibitory concentration (IC_50_) was calculated.

Percentage of Cell viability calculation formula

% Cell viability = (Absorbance of sample)/(Absorbance of control) × 100% (1)

The 50% inhibitory concentration (IC_50_) value is calculated from the graph of the sigmoidal dose–response curve

(2) $$Y=Bottom+\frac{\left(Top-Bottom\right)}{{1+10}^{\left({\log IC}_{50}-X\right)}}$$

where:

Y = Response (e.g., cell viability percentage)
Bottom = Minimum response (e.g., 0% inhibition)
Top = Maximum response (e.g., 100% inhibition)
X = The testing concentration in logarithm unit.
IC_50_ = Concentration at which 50% inhibition occurs

### 3.4. Construction of Metabolic Network Model of L. fermentum KUB-D18 Strain KUB-D18

The genomic sequence data of *L. fermentum* KUB-D18 were obtained from the NCBI database in protein FASTA format. These data were subsequently imported into the ModelSEED platform, a bioinformatics resource that facilitates the mapping of enzymes and genes associated with metabolic reactions sharing common metabolites, both as substrates and products. The gene sequences of the target organism were compared against reference genes in the database, and a preliminary draft of the metabolic model was generated and exported in SBML (.sbml) format. This file represents an initial metabolic model containing a stoichiometric matrix, which enables the systematic analysis of potential biochemical reactions within the cell. The resulting stoichiometric matrix was then employed in flux balance analysis (FBA) to investigate the metabolic capabilities and constraints of the organism.

#### 3.4.1. Gap-Filling of Incomplete Metabolic Pathways in the Genome-Scale

To ensure the completeness and functionality of the genome-scale metabolic model, gap-filling was performed to address potential missing reactions resulting from unidentified or unannotated genes. This process was crucial for restoring the continuity of metabolic pathways, particularly those involved in the biosynthesis of essential metabolites. Two complementary gap-filling strategies were employed: (1) automated gap-filling using the COBRA toolbox, and (2) manual gap-filling guided by curated reactions and metabolites from the ModelSEED and KEGG databases. These approaches collectively ensured the construction of a more comprehensive and biologically consistent genome-scale metabolic model.

#### 3.4.2. Flux Balance Analysis (FBA) and Identification of Key Reactions for Biomass Production in the Genome-Scale Metabolic Model

The reconstructed genome-scale metabolic model of the probiotic bacterium was analyzed using flux balance analysis (FBA) to simulate intracellular reaction fluxes under steady-state conditions. Essential reactions within the metabolic network were identified to determine the key steps required for cell viability and growth. The model was further employed to evaluate the bacterium’s capacity to synthesize specific metabolites and accumulate biomass under various carbon sources. All simulations and analyses were conducted using MATLAB (version R2022b) integrated with the COBRA Toolbox and the Gurobi Optimizer, ensuring computational accuracy and reproducibility.

### 3.5. Predict Biomass Production and LL-37 Peptide from Genome-Scale Metabolic Model

The genome-scale metabolic model of the probiotic bacterium was analyzed in two configurations: (1) a baseline model without the LL-37 peptide biosynthesis pathway, and (2) a modified model incorporating the LL-37 biosynthetic pathway. Both models were evaluated under various carbon-limited conditions to predict biomass accumulation and LL-37 peptide production. The rates of biomass formation and LL-37 peptide synthesis were calculated according to Equations (3) and (4), respectively.

(3) $$Y_{\frac{X}{s}}=\frac{X-X_{0}}{S}$$

where:

$Y_{\frac{X}{s}}$ = Biomass yield (gDW/mmol)
$X_{o}$ = Flux rate of biomass reaction from the intermediate metabolic model (gDW/gDW^−1^h^−1^)
S = Flux rate of the utilized carbon source reaction (mmol/gDW^−1^/h^−1^)
  (4) $$Y_{p}=\frac{P}{S}$$
$Y_{p}$ = LL-37 peptide production rate
P = Flux rate of the LL-37 peptide biosynthesis reaction (mmol/gDWh^−1^)
S = Flux rate of the utilized carbon source reaction (mmol/gDW^−1^h^−1^)

### 3.6. Transform Plasmid and Expression of LL-37 by L. fermentum KUB-D18

The recombinant plasmid pSIP411 + LL-37, containing the coding sequence of LL-37, was constructed based on the pSIP411 vector. To enable detection of secreted LL-37 from *L. fermentum* KUB-D18, a C-terminal His-tag consisting of six histidine residues was appended to the peptide sequence. The plasmid pSIP411 + LL-37 was introduced into *L. fermentum* KUB-D18 via electroporation, using 1000 ng/µL of plasmid DNA per 80 µL of competent cells. The DNA–cell mixture was transferred into a 2 mm gap electroporation cuvette, and a short electrical pulse (2.5 kV, 100 Ω, 25 µF) was applied using a Bio-Rad Gene Pulser (Bio-Rad, Hercules, CA, USA) [37,38].

Immediately after electroporation, 900 µL of MRS medium was added, and the cells were incubated at 37 °C without shaking for 4 h. Transformants harboring pSIP411 + LL-37 were subsequently cultured in MRS medium supplemented with 5 µg/mL erythromycin. Expression of LL-37 was induced by adding 5 ng/mL IP-673 to the culture medium.

### 3.7. Indirect Enzyme-Linked Immunosorbent Assay (ELISA)

The expression of LL-37 peptide by recombinant *L. fermentum* KUB-D18 was quantified using an indirect enzyme-linked immunosorbent assay (ELISA). Briefly, 96-well microtiter plates were coated with 50 µL of conditioned medium or peptide standards and incubated at 4 °C for two nights to facilitate antigen adsorption. After washing with phosphate-buffered saline containing 0.05% Tween-20 (PBS-T), the wells were blocked with 1% bovine serum albumin (BSA) in PBS for 60 min at room temperature to prevent nonspecific binding.

Subsequently, the plates were incubated with anti-His tag primary antibody (1:1000) overnight at 4 °C, followed by three washes with PBS-T. A secondary antibody, goat anti-mouse IgG (1:1000), was then applied and incubated for 60 min at room temperature. After final washing, 3,3′,5,5′-tetramethylbenzidine (TMB) substrate solution (Thermo Fisher Scientific, Waltham, MA, USA) was added and incubated for 10 min. The enzymatic reaction was terminated with 1 M HCl, and absorbance was measured at 450 nm using a microplate reader (BioTek, Winooski, VT, USA).

### 3.8. Statistical Analysis

Statistical analyses were performed to compare multiple groups, including different cell lines, extract concentrations, and time points. Data were analyzed using one-way analysis of variance (ANOVA), followed by Student’s *t*-test and Tukey’s multiple comparison test where appropriate. Results are presented as mean ± standard deviation (SD). Differences were considered statistically significant at *p* < 0.05.

## 4. Conclusions

In this study, we successfully constructed the first genome-scale metabolic model (GEM) of *Lactobacillus fermentum* KUB-D18 and further optimized the model by integrating the biosynthetic pathway of the anticancer peptide LL-37. This model enables accurate simulation of bacterial growth and peptide production under various carbon sources, providing predictive insights into optimal culture conditions. The plasmid-based expression system was validated through experimental assays, demonstrating the secretion of LL-37 into the culture medium and confirming the high accuracy of the in silico predictions.

The integration of computational modeling with experimental validation highlights the power of GEMs in bridging synthetic biology and functional applications. This initial version of the GEM is not only a technical milestone but also a versatile platform for future studies on probiotic-based peptide delivery systems. By guiding experimental design, GEMs can significantly reduce trial-and-error steps, shorten experimental timelines, lower costs, and avoid infeasible laboratory conditions.

Overall, this work demonstrates the feasibility and impact of combining metabolic modeling with laboratory validation to establish a rational framework for probiotic engineering. The model developed here will serve as a valuable resource and reference for researchers interested in metabolic engineering, probiotic biotechnology, and synthetic biology, paving the way for advanced applications in health and therapeutic peptide delivery.

## Figures and Tables

**Table 1:** Sequences of eight Cathelicidin-derived anticancer peptides evaluated in this study.

Peptide Name	Sequence of Peptides
LL-38	ALLGDFFRKSKEKIGKEFKRIVQRIKDFLRNLVPRTES
LL-37	LLGDFFRKSKEKIGKEFKRIVQRIKDFLRNLVPRTES
RK-31	RKSKEKIGKEFKRIVQRIKDFLRNLVPRTES
KS-30	KSKEKIGKEFKRIVQRIKDFLRNLVPRTES
KR-20	KRIVQRIKDFLRNLVPRTES
FK-16	FKRIVQRIKDFLRNLV
FK-13	FKRIVQRIKDFLR
KR-12	KRIVQRIKDFLR

**Table 2:** Prediction analysis of allergenicity using AllerTop, AllergenFP, and AllerCatPro, classified according to probabilistic scores and predicted functions.

Allergenicity Prediction		AL-38	LL-37	RK-31	KS-30	KR-20	FK-16	FK-13	KR1-2
AllerTop	allergen	allergen	None	allergen	allergen	None	None	None	None
AllergenFP	allergen	None	None	None	None	None	None	None	None
AllerCatPro	evidence	None	None	None	None	None	None	None	None

**Table 3:** Prediction analysis of toxicity using ToxinPred and ToxIBTL, classified according to probabilistic scores and predicted functions.

Toxicity Prediction		AL-38	LL-37	RK-31	KS-30	KR-20	FK-16	FK-13	KR1-2
ToxinPred	SVM score	−1.45	−1.58	−1.45	−1.48	−1.64	−1.25	−1.12	−1.28
	Prediction	Non-toxic	Non-toxic	Non-toxic	Non-toxic	Non-toxic	Non-toxic	Non-toxic	Non-toxic
ToxIBTL	Prediction	Non-toxic	Non-toxic	Non-toxic	Non-toxic	Non-toxic	Non-toxic	Non-toxic	Non-toxic

**Table 4:** Prediction analysis of hemolytic activity using HAPPENN, HemoPred, and Macrel, classified according to probabilistic scores and predicted functions.

Hemolytic Prediction	AL-38	LL-37	RK-31	KS-30	KR-20	FK-16	FK-13	KR1-2
HAPPENN- nTer	None	None	None	None	None	None	None	None
HAPPENN- cTer	None	None	None	None	None	None	None	None
HAPPEN- PROB	0.467	0.258	0.008	0.006	0.003	0.192	0.046	0.011
HemoPred	hemolytic	hemolytic	hemolytic	hemolytic	None	hemolytic	hemolytic	hemolytic
Macrel	hemolytic	hemolytic	hemolytic	hemolytic	None	None	None	hemolytic

**Table 5:** IC_50_ values of LL-37, FK-16, and DMSO, assessed on three different cell lines using the MTT assay.

Cell Line	Calculated IC_50_ (µM)								
	PCS-201-010			SW-620			HT-29		
Time/Treatment	24 h	48 h	72 h	24 h	48 h	72 h	24 h	48 h	72 h
LL-37 (µM)	38.72 ± 7.65	42.57 ± 9.02	45.53 ± 5.22	21.42 ± 11.44	15.28 ± 6.67	17.33 ± 8.20	>50.00	>50.00	>50.00
FK-16 (µM)	>50.00	>50.00	>50.00	>50.00	>50.00	>50.00	>50.00	>50.00	>50.00
DMSO (Percentage)	4.65 ± 1.34	2.19 ± 0.81	1.57 ± 0.35	3.52 ± 0.97	2.13 ± 0.49	1.25 ± 0.30	5.48 ± 1.69	3.21 ± 0.77	2.57 ± 0.89

**Table 6:** Comparative analysis of metabolic network characteristics of *L. fermentum* KUB-D18 (*i*TM505) with various bacterial strains, adapted from Luo et al. (2021) [13].

Model	*i*HL622	*i*NF517	LbReuteri	*i*BT721	*i*ML1515	*i*TM505
Organism	*L. reuteri* ATCC PTA 6475	*L. lactis* MG1363	*L. reuteri* JCM 1112	*L. plantarum* WCFS1	*E. coli* MG1655	*L. fermentum* KUB-D18
Genes	2019	2339	1943	3063	4243	1983
Included	622 (31%)	516 (22%)	530 (27%)	724 (24%)	1516 (36%)	505 (25.47%)
Reactions	869	754	714	778	2712	1095
Internal	644	530	507	538	1548	932
Transport	122	119	123	127	833	80
Exchange	103	105	84	113	331	83
Metabolites	713	650	660	662	1877	1191
Biomass consistency	1.00	0.83	-b	-b	1.00	1.00

## Data Availability

The original contributions presented in this study are included in the article. Further inquiries can be directed to the corresponding authors.
